# Effects of 12-month combined aerobic and resistance training on physical fitness, cardiometabolic health and quality of life in middle-aged adults with overweight/obesity at high risk of metabolic syndrome in Poland

**DOI:** 10.1016/j.pmedr.2026.103531

**Published:** 2026-06-06

**Authors:** Piotr Soszyński, Artur Białkowski, Paweł Żochowski, Urszula Religioni

**Affiliations:** a Medicover Sp. z o.o., 00-807 Warsaw, Poland; b School of Public Health, Centre of Postgraduate Medical Education of Warsaw, 01-813 Warsaw, Poland

**Keywords:** Physical activity, Structured exercise, Primary prevention, Body composition, Insulin resistance, Cardiovascular risk, QALY, Obesity

## Abstract

**Objective:**

Physical inactivity is a major modifiable driver of cardiometabolic disease and functional decline. Long-term, real-world evidence on structured exercise in at-risk working-age adults remains limited.

**Methods:**

In this 12-month prospective intervention, 192 overweight or obese, professionally active adults (mean age 46.2 ± 7.2 years; BMI 30.04 ± 2.78 kg/m^2^; 137 men, 55 women) completed a supervised aerobic–resistance program delivered in outpatient and fitness settings between March 2023 and May 2024 in Poland. Outcomes included Cooper test performance, anthropometry and body composition, metabolic markers including insulin resistance HOMA-IR, HeartScore2 10-year cardiovascular mortality risk, health-related quality of life HRQoL, and quality-adjusted life years QALYs.

**Results:**

Cooper distance increased by ∼33% (*p* < 0.01). BMI and body weight decreased modestly, while central adiposity improved more markedly (Body Roundness Index −7.3%; visceral fat rating reduced). HOMA-IR fell by ∼18%, triglycerides and uric acid decreased, HDL-cholesterol increased, and blood pressure declined by ∼3%. HeartScore2 decreased from 4.14% to 3.59% (−13.3%; *p* < 0.01). HRQoL utility improved from 0.66 to 0.76, corresponding to an estimated 1.04 QALY gain.

**Conclusions:**

A 12-month structured physical activity improved cardiometabolic risk factors and QALYs, supporting its role in primary prevention and health-risk reduction for high-risk working-age adults.

## Highlights

• Real-world evidence on long-term structured exercise remains limited.
• Twelve-month exercise improved physical fitness in at-risk adults by 30%.
• Training reduced body mass, central adiposity and visceral fat.
• Insulin resistance, lipid profile and blood pressure improved.
• Cardiovascular risk fell by 13% with about 1.0 quality-adjusted life year gained.

## 1 Introduction

Physical inactivity is a major modifiable risk factor for non-communicable diseases (NCDs), including obesity, cardiovascular disease, type 2 diabetes and some cancers. Sedentary behavior also contributes to reduced functional capacity, impaired mental health and diminished quality of life, creating a substantial burden on healthcare systems and societies (Ding et al., 2016; Katzmarzyk et al., 2022). In contrast, regular physical activity favorably modifies body composition and cardiometabolic risk, improves vascular function and insulin sensitivity, and is associated with lower morbidity and premature mortality (Posadzki et al., 2020; Batrakoulis et al., 2022). Sustained physical activity may also delay age-related decline by mitigating sarcopenia, central obesity and metabolic dysfunction, thereby helping maintain independence and work ability into older age (Li et al., 2023; Verheggen et al., 2016a).

Structured exercise programs are an important tool in preventive and lifestyle medicine (Haidar and Horwich, 2023; Singh et al., 2024). Compared with brief lifestyle advice, supervised programs generally achieve higher adherence and greater improvements in cardiorespiratory fitness, blood pressure, lipid abnormalities and insulin resistance, while also improving mental health, sleep quality and perceived stress (Białkowski et al., 2024a). Longer-duration interventions may be particularly effective in stabilizing behavioral change and consolidating physiological adaptations, but real-world data from working-age adults with elevated cardiometabolic risk remain limited (Lear et al., 2017; Mari et al., 2025).

In our previous six-month study among adults with overweight and obesity, a structured physical activity program led to clinically meaningful reductions in BMI, fat mass and visceral fat, together with improved fitness (Białkowski et al., 2024b). These findings supported the value of medium-duration interventions, but questions remained about additional gains with longer participation.

The present 12-month study extends that work by lengthening the intervention, broadening clinical outcomes and quantifying long-term health gains using quality-adjusted life years (QALYs) in professionally active adults with overweight or obesity. We aimed to provide practice-oriented evidence on how sustained physical activity can reduce health risks, support healthy aging and inform preventive strategies in healthcare and workplace settings.

## 2 Methods

### 2.1 Study design and population

This 12-month prospective intervention study was conducted between March 2023 and May 2024 in Warsaw and Wrocław, Poland. The program was delivered in outpatient medical centers and cooperating fitness facilities, with physicians, nurses and physical activity trainers collaborating in a real-world preventive setting. Participants underwent baseline screening, completed a structured physical activity program and attended periodic medical and fitness assessments. An age-, and BMI-matched comparison group was retrospectively drawn from the same medical database and used only as a descriptive real-world reference, not as a formal control group.

The target population comprised professionally active adults with overweight or stage I obesity and at least one cardiometabolic risk factor. Inclusion criteria were: age ≥ 30 years; BMI 26–35 kg/m^2^ documented in the previous 12 months; at least one of total cholesterol ≥5.70 mmol/L, fasting glucose ≥5.56 mmol/L, or blood pressure ≥ 140/90 mmHg; and no regular physical activity during the preceding 12 months.

Exclusion criteria were conditions limiting moderate-to-vigorous exercise or posing unacceptable risk, including advanced diabetes, uncontrolled hypertension, chronic heart disease, asthma or COPD, active/recent cancer, pregnancy, major musculoskeletal limitations, or any physician-determined contraindication. Of 1155 eligible individuals in electronic medical records, 290 entered recruitment and 240 met all criteria, provided written informed consent and started the program.

The study was conducted as a research experiment under the Act of December 5, 1996 on the professions of doctor and dentist [Journal of Laws 2023.0.1516, Poland], chapter 4. The protocol, patient information, consent template and insurance were approved by Bioethical Committees at the Regional Chambers of Physicians in Warsaw and Wrocław. All participants gave written informed consent, including GDPR-compliant personal data processing. The study was registered in the Open Science Framework database: DOI 10.17605/OSF.IO/NX4MW.

### 2.2 Measures

Recruitment involved electronic-record identification, telephone invitation by a trained nurse and three baseline visits. During the physician visit, a family or internal medicine specialist confirmed eligibility, obtained medical history, performed physical examination and resting ECG, estimated 10-year cardiovascular mortality risk using HeartScore2 (SCORE2 working group and ESC Cardiovascular risk collaboration, 2021), discussed study risks and benefits, and obtained consent.

A physical activity trainer assessed exercise capacity using the 12-min Cooper endurance test (Cooper, 1968) on a treadmill with 0% incline and individually adjusted speed. Additional tests included the Thomayer flexibility test and core stability plank test. Back pain was rated on a 0–10 visual analogue scale.

During the nurse visit, anthropometry measures included height, weight, BMI, waist and hip circumference, waist–hip ratio and Body Roundness Index (Thomas et al., 2013). Body composition was assessed using a multifrequency bioimpedance scale (Tanita) (Ritchie et al., 2005). Blood pressure and heart rate were recorded after standardized rest. Fasting blood samples were analyzed in an accredited laboratory for lipids, glucose, insulin with HOMA-IR (Sharma et al., 2020), liver enzymes, creatinine and uric acid. HRQoL was assessed using WHOQOL-BREF (WHOQOL Group et al., 2004).

The intervention was a 12-month structured physical activity program supervised by qualified trainers. Training combined aerobic and resistance components and followed World Health Organization recommendations: 150–300 min/week of moderate-intensity or 75–150 min/week of vigorous-intensity activity, or an equivalent combination (WHO, 2020). Sessions were individual or small-group based, with type and intensity tailored to baseline fitness, comorbidities and preferences. Trainers provided feedback and behavioral support to encourage adherence.

The program followed general FITT principles; most participants completed 3–5 sessions/week, usually lasting 45–75 min. Intensity was individually prescribed and progressed over time, targeting approximately 60–85% of maximum heart rate. Participants were encouraged to use a Myzone heart-rate wristband to monitor duration, intensity zones and energy expenditure; these data were used exploratorily to assess associations between training intensity and outcomes. Because of the real-world design, training was individualized rather than standardized.

Follow-up assessments at approximately 3, 6 and 9 months included repeated Cooper and fitness tests to monitor progress and adjust training plans. At 12 months, baseline assessments were repeated using the same procedures, including anthropometry, body composition, blood pressure, fitness tests, fasting laboratory parameters, WHOQOL-BREF and HeartScore2.

The comparison group comprised 305 adults matched by age, sex distribution and baseline BMI, who did not participate in the program and continued usual care. BMI, lipids, fasting glucose and blood pressure were available at two time points approximately 12 months apart. Because of its observational, non-randomized design and limited variables, this group was used only for descriptive comparison.

### 2.3 Statistical analysis

Descriptive statistics were expressed as mean, standard deviation, median, minimum and maximum for numerical variables, and counts and percentages for categorical variables. Between-group comparisons used Fisher's exact or Pearson's chi-square tests for categorical variables and Wilcoxon rank-sum or Kruskal–Wallis tests for continuous variables. Pre–post comparisons used paired Student's *t*-tests or Wilcoxon signed-rank tests, depending on Shapiro–Wilk normality testing, and McNemar's test for paired categorical data. Analyses were performed overall and by sex, age and baseline BMI; body-composition parameters with sex-specific norms were analyzed separately in women and men. Formal intervention-versus-comparison analyses were not performed.

Exploratory correlations between body-composition changes and training intensity, defined as time spent in higher heart-rate zones from Myzone data, were assessed using Pearson coefficients. Analyses used available data for each outcome without imputation.

For the 192 completers, a detectable-effect calculation for paired analyses with two-sided alpha 0.05 and 80% power showed that the study could detect a standardized within-person effect size of approximately 0.20. Using baseline standard deviations as conservative proxies for within-person change variability, this corresponded to detectable changes of approximately 83 m in Cooper distance and 0.56 kg/m^2^ in BMI. Thus, the sample size was sufficient to detect prespecified clinically meaningful improvements of 10% in Cooper distance and 2% in BMI.

Clinical effect sizes were expressed as absolute and relative changes from baseline to 12 months. Standardized paired effect sizes were calculated as standardized response means, defined as mean within-person change divided by the standard deviation of individual change scores.

HRQoL measured with WHOQOL-BREF at baseline and 12 months was transformed into utility values. QALYs were estimated as utility × time over a 10-year horizon, assuming maintenance of the 12-month gain (Drummond et al., 2015). Ten-year cardiovascular mortality risk was estimated with HeartScore2; absolute risk reduction was converted into expected life-years gained and multiplied by post-intervention utility to estimate additional QALYs from reduced cardiovascular mortality. These modelled estimates should be interpreted cautiously.

Analyses were performed with R statistical computing software (version 4.4.1 or later).

## 3 Results

### 3.1 Participant baseline characteristics and physical fitness

Of the 240 participants who entered the program, 192 (137 men, 55 women) completed the 12-month intervention and the full set of follow-up assessments, so the drop-out rate was 20%. Their mean age was 46.2 ± 7.2 years; women were slightly older than men (48.7 ± 7.6 vs. 45.2 ± 6.8 years). There was no statistically significant difference in BMI between males and females at baseline (29.92 ± 2.87 vs. 30.35 ± 2.53; *p* = 0.22). Overweight (BMI 26–29.9 kg/m^2^, *n* = 99) and obese (BMI 30–35 kg/m^2^, *n* = 93) participants were both well represented, and baseline BMI did not differ significantly by sex.

The Cooper test was used to assess physical fitness before and after the 12-month intervention. Among all participants, the mean distance increased by almost 33% from 1491.2 ± 409.5 m at baseline to 1980.5 ± 455.0 m after 12 months (*p* < 0.01). Males improved by 33.7% from 1594.3 ± 416.0 m to 2132.2 ± 413.0 m, while females improved by 30% from 1231.1 ± 247.2 m to 1603.0 ± 315.2 m (both p < 0.01), with no statistical significant difference between sexes. Majority (71%) of subjects achieved 20% or greater improvement in Cooper test. Participants with an initial BMI below 30 showed greater improvements than those with a BMI ≥30, although both groups demonstrated statistically significant gains. Improvements were also observed across all age categories, with the greatest performance levels achieved in younger age groups.

In addition, the back pain index, measured on a visual analog scale (VAS) ranging from 1 (no pain) to 10 (most severe pain), demonstrated a significant reduction following the 12-month training period for all participants, with initial mean score 1.9 ± 2.2 compared to 0.7 ± 1.4 at the end (*p* < 0.01). Moreover, other physical fitness tests (Thomayer fingers-to-floor, plank) showed significant enhancements after the training.

### 3.2 Anthropometry, body composition, metabolic and biochemical markers

Anthropometric indices changed favorably over the 12 months (Table 1). Mean body weight decreased from 91.8 ± 12.4 kg to 89.4 ± 12.3 kg (*p* < 0.01), and BMI from 30.04 ± 2.78 to 29.26 ± 2.93 kg/m^2^ (*p* < 0.01). Similar relative reductions were seen in men and women. Waist circumference and, to a lesser degree, hip circumference declined significantly, leading to a modest but significant reduction in waist–hip ratio in the overall cohort.

**Table 1:** Anthropometric measures at baseline and after 12-month structured training in total population and gender subgroups of overweight and obese adults in Poland (2023–2024).

Analyzed Measure/Group	Baseline			After 12-month Training			*p-*Value^1^
	Mean (SD)	Median [IQR]	Minimum, Maximum	Mean (SD)	Median [IQR]	Minimum, Maximum	
**Weight, kg**							
All (*N* = 192 → 160)	91.8 (12.4)	90.3 [82.8, 100.6]	64, 127	89.4 (12.3)	88.5 [81.7, 98.9]	62, 122	<0.01
Males (*N* = 135 → 112)	95.6 (11.5)	94.7 [87.0, 105.7]	69, 127	93.3 (11.5)	92.0 [84.4, 101,0]	69, 122	<0.01
Females (*N* = 55 → 48)	82.5 (9.2)	82.6 [77.0, 86.7]	64, 107	80.1 (8.9)	81.8 [73.8, 85.4]	62, 102	0.01
**BMI, kg/m**^**2**^							
All (N = 192 → 159)	30.04 (2.78)	29.77 [27.79, 32.12]	24.5, 37.9	29.26 (2.93)	28.67 [27.06, 31.04]	23.7, 37.1	<0.01
Males (*N* = 137 → 112)	29.91 (2.88)	29.63 [27.56, 32.09]	24.5, 37.9	29.24 (2.99)	28.62 [27.07, 31.04]	23.7, 37.1	<0.01
Females (N = 55 → 47)	30.35 (2.53)	30.05 [28.64, 32.25]	25.0, 36.2	29.32 (2.83)	29.76 [27.16, 31.02]	24.1, 35.5	0.01
**Waist circumference, cm**							
All (*N* = 189 → 150)	100.3 (9.4)	100.0 [93.0, 106,0]	80, 126	97.2 (10.2)	96.5 [90.0, 104.0]	83, 124	<0.01
Males (N = 135 → 105)	103.4 (8.3)	103.0 [97.8, 109.0]	85, 126	100.3 (9.7)	100.0 [93.0, 107.0]	76, 126	<0.01
Females (*N* = 54 → 45)	92.5 (7.1)	91.0 [87.5, 96.0]	80, 118	89.9 (7.5)	90.0 [84.0, 95.0]	74, 106	0.03
**Hip circumference, cm**							
All (N = 189 → 149)	108.1 (7.2)	108.0 [103.3, 113.0]	92, 128	105.8 (8.0)	106.0 [100.0, 112.0]	83, 124	<0.01
Males (*N* = 136 → 104)	107.2 (6.8)	107.0 [102.0, 111.6]	92, 128	105.0 (7.6)	105.0 [99.0, 110.0]	90, 121	<0.01
Females (N = 54 → 45)	110.3 (7.6)	109.0 [105.1, 115.8]	96, 126	107.7 (8.7)	108.0 [103.0, 114.0]	83, 124	NS
**WHR**							
All (*N* = 190 → 149)	0.929 (0.088)	0.900 [0.900, 1.000]	0.68, 1,11	0.917 (0.091)	0.900 [0.880, 1.000]	0.71, 1.07	0.04
Males (N = 135 → 104)	0.964 (0.059)	0.960 [0.930, 1.000]	0.81, 1.11	0.956 (0.063)	0.960 [0.910, 1.000]	0.72, 1.07	NS
Females (N = 54 → 45)	0.840 (0.061)	0.835 [0.800, 0.880]	0.68, 0.98	0.838 (0.070)	0.830 [0.790, 0.890]	0.71, 0.99	NS
**BRI**							
All (N = 189 → 150)	4.90 (1.03)	4.69 [4.15, 5.56]	2.8, 7.8	4.54 (1.10)	4.41 [3.73, 5.30]	2.0, 7.9	<0.01
Males (N = 135 → 105)	5.01 (1.04)	4.95 [4.20, 5.68]	2.8, 7.8	4.66 (1.15)	4.40 [3.89, 5.33]	2.0, 7.9	<0.01
Females (N = 54 → 45)	4.62 (0.95)	4.34 [4.03, 5.00]	2.8, 7.7	4.26 (0.93)	4.42 [3.47, 4.91]	2.5, 6.0	<0.03

IQR – interquartile range, BMI – body mass index, WHR – waist-hip ratio, BRI – body roundness index. ^1^ Paired *t-*test.

Body Roundness Index (BRI), a marker of central adiposity and cardiometabolic risk, fell from 4.90 ± 1.03 to 4.54 ± 1.10 (p < 0.01), with comparable changes in both sexes. The relative decline of 7.3% in BRI was the largest among the anthropometric measures, indicating a preferential reduction in abdominal adiposity.

Body composition improved meaningfully (Table 2). Fat mass decreased significantly in men (from 24.49 ± 6.63 kg to 22.98 ± 6.91 kg; *p* = 0.01) and showed a strong trend in women (from 30.59 ± 5.95 kg to 28.65 ± 5.58 kg; *p* = 0.05). Visceral fat rating decreased in both men (11.6 ± 2.9 to 11.0 ± 3.1; p = 0.01) and women (8.5 ± 1.9 to 7.8 ± 1.7; *p* = 0.03). The fat mass to fat-free mass (FM/FFM) ratio improved significantly in men, and percentage muscle mass (%MM) increased in men, while absolute muscle mass remained stable. No clinically relevant changes were observed in total body water or absolute fat-free mass.

**Table 2:** Body composition values and metrics at baseline and after 12-month training in total population and gender subgroups of overweight and obese adults in Poland (2023–2024).

Analyzed Measure/Group	Baseline			After 12-month Training			*p-*Value^1^
	Mean (SD)	Median [IQR]	Minimum, Maximum	Mean (SD)	Median [IQR]	Minimum, Maximum	
**Fat mass (FM), kg**							
Males (N = 136 → 106)	24.5 (6.6)	24.1 [19.7, 28.7]	12.0, 41.4	23.0 (6.91)	21.8 [18.1, 27.9]	8.5, 42.3	<0.01
Females (N = 55 → 45)	30.6 (6.0)	29.8 [27.2, 33.7]	14.3, 46.5	28.7 (5.6)	28.2 [24.7, 32.8]	18.7, 42.5	0.05
**%FM**							
Males (N = 136 → 106)	25.2 (4.4)	25.0 [22.2, 28.2]	14.8, 38.0	24.1 (4.8)	23.6 [20.9, 27.9]	12.0, 35.3	<0.01
Females (N = 55 → 47)	36.9 (4.2)	37.4 [34.9, 39.2]	17.1, 44.4	35.7 (3.8)	36.2 [32.8, 38.5]	26.4, 42.7	NS
**Visceral Fat Rate (VFR)**							
Males (N = 136 → 103)	11.6 (2.9)	11.0 [9.0, 13.0]	6, 19	11.0 (3.1)	11.0 [9.0, 13.0]	4, 19	<0.02
Females (N = 54 → 43)	8.5 (1.9)	8.0 [7.0, 10.0]	4, 13	7.8 (1.7)	8.0 [7.0, 9.0]	4, 11	0.03
**FM/FFM**							
Males (N = 135 → 104)	0.34 (0.08)	0.34 [0.29, 0.39]	0.17, 0.61	0.32 (0.08)	0.31 [0.26, 0.39]	0.14, 0.55	<0.01
Females (N = 55 → 45)	0.59 (0.10)	0.60 [0.54, 0.65]	0.21, 0.80	0.56 (0.09)	0.57 [0.49, 0.62]	0.36, 0.74	NS
**Muscle Mass (MM), kg**							
Males (N = 136 → 106)	67.6 (5.9)	68.0 [62.8, 71.5]	52.9, 86.6	67.0 (5.9)	66.5 [62.6, 71.3]	53.4, 83.0	NS
Females (N = 54 → 45)	49.3 (5.0)	49.6 [45.7, 51.9]	39.7, 65.6	48.5 (4.5)	48.7 [45.3, 51.2]	38.6, 58.5	NS
**%MM**							
Males (*N* = 134 → 106)	71.1 (4.1)	71.2 [68.2, 73.9]	58.9, 81.0	72.1 (4.6)	72.7 [68.5, 75.2]	61.5, 83.6	<0.01
Females (N = 55 → 45)	59.9 (4.0)	59.4 [57.7, 61.9]	52.8, 78.7	61.1 (3.6)	60.5 [58.4, 63.9]	54.5, 69.8	NS

IQR – interquartile range, ^1^ Paired *t-*test.

Exploratory analyses combining Myzone training data with changes in body composition showed that reductions in BMI, fat mass, percentage fat mass and visceral fat were moderately correlated with time spent in higher heart-rate zones (>60% of HRmax), with correlation coefficients around 0.2–0.3 (*p* < 0.05). Increases in %MM showed similar positive correlations with higher-intensity training time, suggesting that training intensity contributed to favorable body-composition changes.

Metabolic and biochemical parameters improved in parallel with changes in fitness and body composition (Table 3). Fasting glucose and insulin decreased significantly, with HOMA-IR declining from 2.95 ± 1.66 to 2.51 ± 1.67 in men and from 2.63 ± 1.34 to 1.96 ± 1.00 in women (both *p* < 0.01), indicating improved insulin sensitivity.

**Table 3:** Laboratory results at baseline and after 12-month training in total population and gender subgroups of overweight and obese adults in Poland (2023–2024).

Analyzed Measure/Group	Baseline			After 12-month Training			*p-*Value^1^
	Mean (SD)	Median [IQR]	Minimum, Maximum	Mean (SD)	Median [IQR]	Minimum, Maximum	
**ALT, ukat/L**							
All (N = 192 → 160)	0.55 (0.36)	0.47 [0.35, 0.68]	0.13, 3.76	0.50 (0.21)	0.45 [0.35, 0.60]	0.13, 1.29	<0.03
Males (N = 137 → 112)	0.63 (0.39)	0.52 [0.42, 0.77]	0.20, 3.76	0.54 (0.21)	0.50 [0.40, 0.63]	0.20, 1.29	0.01
Females (N = 55 → 47)	0.35 (0.14)	0.33 [0.26, 0.41]	0.13, 0.82	0.38 (0.14)	0.35 [0.27, 0.47]	0.13, 0.70	NS
**AST, ukat/L**							
All (*N* = 191 → 159)	0.49 (0.40)	0.40 [0.33, 0.51]	0.21, 3.62	0.43 (0.14)	0.40 [0.35, 0.48]	0.20, 1.27	<0.03
Males (N = 136 → 112)	0.55 (0.46)	0.44 [0.37, 0.57]	0.23, 3.62	0.44 (0.11)	0.42 [0.37, 0.50]	0.25, 0.83	<0.01
Females (N = 55 → 46)	0.35 (0.09)	0.34 [0.30, 0.39]	0.21, 0.58	0.40 (0.19)	0.37 [0.30, 0.43]	0.20, 1.27	<0.04
**GTP, ukat/L**							
All (N = 191 → 157)	0.59 (0.54)	0.48 [0.33, 0.68]	0.13, 6.40	0.53 (0.44)	0.42 [0.28, 0.65]	0.12, 4.79	<0.01
Males (N = 135 → 105)	0.68 (0.60)	0.53 [0.43, 0.73]	0.20, 6.40	0.60 (0.49)	0.50 [0.35, 0.71]	0.22, 4.79	<0.01
Females (N = 55 → 47)	0.36 (0.21)	0.30 [0.22, 0.43]	0.13, 1.14	0.36 (0.22)	0.28 [0.22, 0.39]	0.12, 1.02	NS
**Glucose, mmol/l**							
All (N = 191 → 157)	5.30 (0.54)	5.22 [4.95, 5.54]	4.17, 8.61	5.04 (0.58)	5.00 [4.68, 5.28]	3.89, 9.22	<0.01
Males (N = 136 → 104)	5.36 (0.57)	5.25 [4.98, 5.58]	4.42, 8.61	5.09 (0.62)	5.06 [4.70, 5.28]	3.89, 9.22	<0.01
Females (N = 55 → 48)	5.16 (0.43)	5.11 [4.87, 5.40]	4.17, 6.68	4.94 (0.47)	4.89 [4.67, 5.25]	4.11, 5.94	<0.01
**Insulin, pmol/l**							
All (N = 190 → 149)	71.7 (36.1)	63.3 [45.6, 88.8]	22.1, 214.8	61.3 (34.6)	52.1 [35.9, 75.6]	16.0, 225.6	<0.01
Males (N = 135 → 104)	73.2 (37.7)	63.8 [46.0, 94.5]	25.6, 214.8	65.1 (37.9)	57.1 [36.5, 83.7]	16.6, 225.6	<0.01
Females (N = 55 → 47)	68,0 (31.6)	62.4 [43.9, 84.0]	22.1, 177.0	52.5 (23.1)	46.1 [36.0, 70.5]	16.0. 130.2	<0.01
**HOMA-IR**							
All (N = 189 → 157)	2.86 (1.60)	2.45 [1.74, 3.48]	0.75, 8.77	2.34 (1.52)	2.01 [1.31, 2.86]	0.51, 11.14	<0.01
Males (N = 134 → 104)	2.95 (1.66)	2.55 [1.76, 3.72]	0.98, 8.77	2.51 (1.67)	2.17 [1.35, 3.12]	0.59, 11.14	<0.01
Females (N = 55 → 47)	2.63 (1.34)	2.25 [1.72, 3.22]	0.75, 7.17	1.96 (1.0)	1.71 [1.28, 2.53]	0.51, 5.57	<0.01
**IGF-1, nmol/l**							
All (*N* = 177 → 150)	16.8 (4.6)	16.2 [13.4, 18.9]	7.5, 31.3	17.3 (4.6)	16.9 [14.0, 19.5]	8.5, 33.9	NS
Males (*N* = 125 → 105)	16.9 (4.8)	16.4 [13.9, 18.7]	7.5, 31.3	17.6 (4.5)	17.5 [14.0, 20.2]	9.0, 28.4	NS
Females (N = 54 → 45)	16.5 (4.2)	16.0 [13.2, 19.2]	8.4, 26.3	16.5 (4.6)	16.1 [14.0, 18.9]	8.5, 33.9	NS
**Total Cholesterol, mmol/l**							
All (N = 192 → 150)	5.49 (1.01)	5.61 [4.79, 6.09]	3.16, 8.70	5.44 (1.06)	5.52 [4.71, 6.16]	2.75, 8.11	NS
Males (N = 137 → 105)	5.50 (1.08)	5.62 [4.74, 6.09]	3.16, 8.70	5.46 (1.11)	5.63 [4.69, 6.20]	2.93, 8.11	NS
Females (N = 55 → 45)	5.46 (0.83)	5.44 [4.90, 6.02]	3.76, 7.72	5.40 (0.92)	5.31 [4.71, 6.06]	2.75, 7.28	NS
**LDL-cholesterol, mmol/l**							
All (*N* = 188 → 150)	3.40 (0.92)	3.40 [2.73, 4.04]	1.06, 5.91	3.33 (0.96)	3.42 [2.67, 3.95]	0.96, 6.09	NS
Males (N = 137 → 105)	3.47 (0.98)	3.46 [2.79, 4.15]	1.06, 5.91	3.39 (1.02)	3.47 (2.64, 4.01)	0.98, 6.09	NS
Females (N = 55 → 45)	3.23 (0.73)	3.16 [2.68, 3.72]	1.76, 5.03	3.20 (0.80)	3.12 [2.74, 3.76]	0.96, 4.95	NS
**HDL-cholesterol, mmol/l**							
All (N = 192 → 150)	1.36 (0.34)	1.30 [1.13, 1.54]	0.62, 2.49	1.43 (0.32)	1.39 (1.19, 1.66)	0.70, 2.38	<0.01
Males (N = 137 → 105)	1.25 (0.27)	1.23 [1.06, 1.40]	0.62, 2.10	1.33 (0.29)	1.30 (1.14, 1.48)	0.70, 2.20	<0.01
Females (N = 55 → 45)	1.63 (0.35)	1.58 (1.42, 1.81]	1.01, 2.49	1.66 (0.27)	1.66 [1.45, 1.84]	1.09, 2.38	NS
**Triglycerides, mmol/l**							
All (N = 192 → 150)	1.67 (1.07)	1.41 [1.04, 1.96]	0.43, 9.38	1.49 (0.90)	1.24 [0.88, 1.78]	0.49, 6.43	<0.01
Males (N = 137 → 105)	1.80 (1.18)	1.49 [1.06, 2.06]	0.50, 9.38	1.63 (0.99)	1.38 [0.91, 2.08]	0.49, 6.43	<0.02
Females (N = 54 → 45)	1.34 (0.65)	1.20 [0.89, 1.76]	0.43, 3.51	1.16 (0.53)	1.05 [0.76, 1.31]	0.62, 3.41	<0.03
**Creatinine, mmol/l**							
All (N = 191 → 150)	75.7 (12.3)	76.0 [67.2, 84.0]	43.3, 112.3	79.9 (12.3)	79.6 [70.7, 86.6]	48.6, 106.1	<0.01
Males (N = 136 → 105)	79.8 (11.0)	78.7 [73.2, 86.6]	46.9, 112.3	84.1 (11.3)	83.1 [78.7, 91.1]	53.9, 106.1	<0.01
Females (N = 54 → 48)	65.5 (9.2)	64.5 [59.7, 71.6]	43.3, 87.5	70.0 (8.4)	69.8 [65.4, 76.0]	48.6, 86.6	<0.01
**Uric Acid, mmol/l**							
All (N = 192 → 150)	353.5 (79.4)	345.0 [297.4, 410.4]	178.4, 553.2	335.4 (73.1)	321.2 [285.5, 377.7]	154.6, 571.0	<0.01
Males (N = 137 → 105)	380.3 (72.3)	374.7 [327.1, 428.3]	232.0, 553.2	356.3 (69.9)	350.9 [301.9, 404.5]	249.8, 571.0	<0.01
Females (N = 54 → 47)	286.7 (52.9)	285.5 [255.8, 318.2]	178.4, 422.3	285.4 (54.2)	297.4 [237.9, 321.2]	154.6, 392.6	NS

IQR – interquartile range, ALT – alanine aminotransferase, AST – aspartate aminotransferase, GTP – Gama glutamyl transpeptidase, HOMA-IR – Homeostatic Model Assessment for Insulin Resistance, IGF-1 – insulin-like growth factor 1, ^1^ Paired *t-*test.

Serum triglycerides and uric acid levels fell significantly in the whole cohort and within both sexes, while HDL-cholesterol increased in men. Total cholesterol, LDL-cholesterol and IGF-1 did not change significantly. Liver enzymes (ALT, AST, GGT) showed modest but statistically significant decreases, predominantly in men, consistent with reduced hepatic and systemic metabolic stress. Creatinine increased slightly but remained within normal limits, likely reflecting increased or preserved muscle mass.

In the age- and BMI-matched comparison group (*n* = 305), which did not receive the physical activity intervention, BMI remained essentially unchanged over the 12-month observation (29.8 ± 2.5 vs. 30.0 ± 3.3 kg/m^2^). Other available parameters showed no meaningful improvement; in fact, small but statistically significant increases were observed in some risk factors, including total cholesterol, fasting glucose and blood pressure. This contrasted with the favorable trajectories seen in the intervention group.

### 3.3 Cardiovascular outcomes and clinical effects

Resting blood pressure and heart rate decreased significantly among participants completing the program. Mean systolic blood pressure fell from 133.9 ± 15.1 to 129.6 ± 14.9 mmHg and diastolic from 88.2 ± 10.1 to 85.3 ± 9.8 mmHg (both *p* < 0.01). Resting heart rate decreased from 71.2 ± 10.5 to 68.3 ± 10.5 beats per minute (p < 0.01).

Among participants aged ≥40 years, estimated 10-year cardiovascular mortality risk (HeartScore2) declined from 4.14 ± 2.61% at baseline (*n* = 162) to 3.59 ± 2.19% at 12 months (*n* = 150; p < 0.01), representing a relative reduction of 13.3%. In those with higher initial risk (HeartScore2 ≥ 5%), risk decreased from 7.30 ± 2.09% (*n* = 51) to 5.93 ± 2.12% (*n* = 47; p < 0.01), a relative reduction of 18.9%.

No serious adverse events were identified during the intervention period.

Clinical effect sizes were calculated as absolute and relative changes from baseline to 12 months for selected key outcomes. The largest standardized effect was observed for Cooper test distance, indicating a marked improvement in cardiorespiratory fitness. Smaller standardized effects were noted for BMI, Body Roundness Index, visceral fat rating, glucose, insulin, HOMA-IR, blood pressure and HeartScore2; however, these changes were clinically relevant given their favorable direction, consistency, and relationship to cardiometabolic risk reduction. Table with absolute and relative changes, paired effect size and 95% CI for change is included in supplement.

### 3.4 Health-related quality of life and QALYs

Health-related quality of life improved significantly across all WHOQOL-BREF domains (Table 4). Participants reported better overall quality of life and health satisfaction, higher energy levels, improved body image, and better ability to perform daily activities. Reductions in pain and discomfort were particularly notable in men, in line with improvements in back pain scores.

**Table 4:** Selected WHOQOL-BREF quality of life scores at baseline and after 12-month training in total population and gender subgroups of overweight and obese adults in Poland (2023–2024).

Analyzed Measure/Group	Baseline			After 12-month Training			*p-*Value^1^
	Mean (SD)	Median [IQR]	Minimum, Maximum	Mean (SD)	Median [IQR]	Minimum, Maximum	
**How would you rate your quality of life?**							
All (*N* = 141)	3.88 (0.72)	4 [4, 4]	2, 5	4.22 (0.73)	4 [4, 5]	1, 5	<0.01
Males (*N* = 97)	3.91 (0.71)	5 [4, 4]	2, 5	4.22 (0.74)	4 [4, 5]	1, 5	<0.01
Females (*N* = 44)	3.81 (0.76)	4 [3.5, 4]	2, 5	4.23 (0.71)	4 [4, 5]	2, 5	<0.01
**How satisfied are you with your health?**							
All (N = 141)	3.29 (0.78)	3 [3, 4]	1, 5	3.80 (0.76)	4 [3, 4]	2, 5	<0.01
Males (N = 97)	3.29 (0.76)	3 [3, 4]	1, 5	3.83 (0.74)	4 [4, 4]	2, 5	<0.01
Females (N = 44)	3.30 (0.82)	4 [3, 4]	2, 4	3.73 (0.82)	4 [3, 4]	2, 5	<0.01
**To what extent do you feel that (physical) pain prevents you from doing what you need to do?**							
All (N = 141)	3.98 (0.99)	4 [3, 5]	1, 5	4.26 (0.84)	4 [4, 5]	1, 5	<0.01
Males (N = 97)	4.02 (0.97)	4 [3, 5]	1, 5	4.35 (0.82)	5 [4, 5]	1, 5	<0.01
Females (N = 44)	3.89 (1.04)	4 [3, 5]	1, 5	4.05 (0.86)	4 [4, 5]	2, 5	NS
**Do you have enough energy for everyday life?**							
All (N = 141)	3.44 (0.81)	4 [3, 4]	1, 5	3.87 (0.87)	4 [3, 4]	1, 5	<0.01
Males (N = 97)	3.51 (0.82)	4 [3, 4]	1, 5	3.89 (0.84)	4 [4, 4]	1, 5	<0.01
Females (N = 44)	3.27 (0.76)	3 [3, 4]	1, 5	3.82 (0.92)	4 [3, 4]	1, 5	<0.01
**Are you able to accept your bodily appearance?**							
All (N = 141)	3.29 (0.94)	3 [3, 4]	1, 5	3.83 (0.85)	4 [3, 4]	1, 5	<0.01
Males (N = 97)	3.42 (0.94)	3 [3, 4]	1, 5	3.89 (0.87)	4 [4, 4]	1, 5	<0.01
Females (N = 44)	3.02 (0.90)	3 [2, 4]	1, 4	3.68 (0.80}	4 [3, 4]	2, 5	<0.01
**How satisfied are you with your sleep?**							
All (N = 141)	3.22 (0.97)	3 [2, 4]	1, 5	3.50 (0.94)	4 [3, 4]	1, 5	<0.01
Males (N = 97)	3.25 (0.99)	3.5 [2, 4]	1, 5	3.43 (1.01)	4 [3, 4]	1, 5	<0.02
Females (N = 44)	3.16 (0.94)	3 [2.8, 4]	1, 5	3.64 (0.78)	4 [3, 4]	2, 5	<0.01
**How satisfied are you with your ability to perform your daily living activities?**							
All (N = 141)	2.99 (0.88)	3 [2, 4]	1, 5	3.81 (0.80)	4 [3, 4]	1, 5	<0.01
Males (N = 97)	3.00 (0.93)	3 [2, 4]	1, 5	3.86 (0.82)	4 [4, 4]	1, 5	<0.01
Females (N = 44)	2.98 (0.76)	3 [2, 4]	2, 4	3.68 (0.74)	4 [3, 4]	1, 5	<0.01

WHOQOL-BREF – World Health Organization Quality of Life – Brief, IQR – interquartile range, ^1^ Paired *t-*test.

Mean utility values derived from HRQoL increased from 0.66 at baseline to 0.76 after 12 months. Extrapolated over a 10-year horizon, this corresponded to an estimated gain of 1.0 QALY per person from improved quality of life alone. The reduction in 10-year cardiovascular mortality risk from 4.14% to 3.59% yielded an additional 0.0418 QALYs per person when converted into expected life-years gained and weighted by post-intervention utility. Overall, the intervention was associated with an estimated incremental gain of approximately 1.04 QALYs per participant relative to baseline risk and quality of life.

## 4 Discussion

In this 12-month prospective intervention among overweight and obese working-age adults, a structured physical activity program was associated with improvements in physical fitness, body composition, metabolic profile, estimated cardiovascular risk and quality of life. By extending our previous 6-month evaluation (Białkowski et al., 2024b) and broadening the set of outcomes, we show that longer-term supervised exercise can deliver sustained, multidimensional health benefits that are relevant for real-world primary preventive practice.

The large improvement in Cooper test performance in both sexes indicates a substantial gain in cardiorespiratory fitness, a strong predictor of cardiovascular and all-cause mortality. Enhanced fitness, better flexibility and core stability, and reduced back pain suggest not only lower disease risk but also improved functional capacity important for healthy aging, autonomy and work ability (Murray et al., 2023; Tari et al., 2025).

Anthropometric and body-composition changes reinforce this picture. While several outcomes, such as weight loss, reached statistical significance, their clinical relevance varied, with more pronounced changes observed in central adiposity, visceral fat, and insulin resistance than in body weight**.** Because central obesity is more strongly linked to insulin resistance, type 2 diabetes and cardiovascular disease than total weight, these patterns are particularly favorable from a preventive standpoint (König et al., 2010; Verheggen et al., 2016b). Preservation or slight improvement of relative muscle mass, especially in men, suggests that the program helped counteract sarcopenic tendencies while reducing fat mass, supporting both metabolic health and physical performance (Srikanthan and Karlamangla, 2014).

Correlations between higher-intensity training and favorable body-composition changes are in line with evidence that both total volume and intensity of physical activity contribute to risk reduction (Coates et al., 2023). This supports recommendations to gradually incorporate more vigorous training, when safe and acceptable, in preventive programs rather than relying on moderate activity.

The intervention produced robust improvements in insulin resistance, with significant reductions in fasting insulin, glucose and HOMA-IR. Exercise-induced enhancement of insulin sensitivity is central to preventing type 2 diabetes and cardiometabolic disease (Ormazabal et al., 2018; Bird and Hawley, 2017). Parallel improvements in triglycerides, HDL-cholesterol, liver enzymes and uric acid indicate attenuation of metabolic and inflammatory stress and suggest a broad cardiometabolic benefit beyond single risk factors (Lin et al., 2015).

Clinically meaningful blood pressure reductions, together with lipid and insulin changes, translated into a 13–19% relative reduction in estimated 10-year cardiovascular mortality, particularly among those at higher baseline risk. Achieving such risk modification through a non-pharmacological, lifestyle-based approach underscores the importance of exercise programs as an important component of primary prevention and an adjunct to pharmacotherapy (Barone Gibbs et al., 2021).

The matched comparison group, which did not participate in the program, showed stable or slightly worsening risk profiles over the same period. However, given the non-randomized design and limited availability of variables, this comparison should be interpreted cautiously and does not allow causal inference regarding the observed differences.

Beyond physiological outcomes, the intervention substantially improved HRQoL across physical, psychological, social and environmental domains. Increased energy, reduced pain, better perceived health and body image, and improved daily functioning are relevant for individual wellbeing and may support long-term adherence to an active lifestyle and maintenance of behavioral change (Marquez et al., 2020).

By translating HRQoL changes and risk reduction into QALYs, we provide an integrated metric widely used in health interventions assessment and priority setting (Noto et al., 2021). An estimated gain of 1.04 QALY per person from improved quality of life plus from reduced cardiovascular mortality risk over 10 years, represents a substantial benefit for a relatively low-cost program based on supervised exercise and existing infrastructure. Although we did not perform a formal cost-effectiveness analysis, the magnitude of QALY gain suggests that such programs are likely to be potentially cost-effective, when offered to high-risk populations at scale (Pinheiro et al., 2023).

Because participants were professionally active adults, the findings are particularly relevant for workplace health promotion and occupational medicine. Improved fitness, metabolic health and quality of life may translate into fewer sick days, better work performance and delayed disability. Integrating structured physical activity into preventive care pathways and workplace initiatives could therefore contribute simultaneously to NCD prevention, productivity and healthy aging in the workforce (Grimani et al., 2019).

From a policy perspective, our results support the potential role of physical activity promotion as a component of NCD-prevention strategies (World Health Organization, 2018). The data illustrate that well-designed supervised programs delivered in routine practice settings may provide benefits complementary to pharmacological approaches in appropriately selected populations.

Strengths of this study include its 12-month duration, relatively large sample of high-risk but community-dwelling adults, and comprehensive assessment of fitness, anthropometry, body composition, metabolic markers, cardiovascular risk and HRQoL. The inclusion of QALY estimates adds health-economic relevance. Real-world implementation in outpatient and fitness settings enhances generalizability to preventive practice and to health systems seeking scalable, non-pharmacological interventions.

This study has several limitations that should be considered when interpreting the findings. The absence of randomization and a formal control group limits the ability to draw causal inferences, despite the inclusion of a matched comparison cohort. Analyses were based on available data without imputation, which may introduce some degree of attrition bias, particularly given varying sample sizes across outcomes. The intervention was individualized and delivered in real-world settings, which, while enhancing practical relevance, may limit precise characterization and replication. Finally, extrapolation of QALY gains over 10 years assumes maintenance of benefits and should be interpreted with caution; longer-term follow-up would be valuable.

## 5 Conclusions

A 12-month structured physical activity program delivered in routine outpatient and fitness settings was associated with improvements in physical fitness, body composition, insulin resistance, cardiovascular risk and health-related quality of life in working-age adults with overweight and obesity. When expressed in QALYs, the intervention suggested a potential gain in expected healthy life years. These findings indicate that structured exercise programs may represent a valuable component of preventive care and workplace health promotion, although further controlled studies are needed to confirm causal relationships and long-term effects.

## CRediT authorship contribution statement

**Piotr Soszyński:** Writing – review & editing, Writing – original draft, Project administration, Methodology, Investigation, Formal analysis, Data curation, Conceptualization. **Artur Białkowski:** Writing – review & editing, Investigation, Funding acquisition, Formal analysis, Data curation, Conceptualization. **Paweł Żochowski:** Writing – review & editing, Investigation, Data curation. **Urszula Religioni:** Writing – review & editing.

## Funding sources

The study was supported by a research grant from the Medicover Poland.

## Declaration of competing interest

The authors declare that they have no known competing financial interests or personal relationships that could have appeared to influence the work reported in this paper.

## Data Availability

All data are available from the corresponding author on justifiable request.

## Appendix A Supplementary data

Supplementary material Clinical effect sizes
