# Supplementary material for: Effects of 12-month combined aerobic and resistance training on physical fitness, cardiometabolic health and quality of life in middle-aged adults with overweight/obesity at high risk of metabolic syndrome in Poland
Source: Prev Med Rep. 2026 Jun 6;67:103531. doi: 10.1016/j.pmedr.2026.103531 (PMC13273465; doi:10.1016/j.pmedr.2026.103531)
Supplement: Supplementary material — Clinical effect sizes [file mmc1.docx]

**Supplement 1. Clinical effect sizes**

Clinical effect sizes were calculated as absolute and relative changes from baseline to 12 months for selected key outcomes. The largest standardized effect was observed for Cooper test distance, indicating a marked improvement in cardiorespiratory fitness. Smaller standardized effects were noted for BMI, Body Roundness Index, visceral fat rating, glucose, insulin, HOMA-IR, blood pressure and HeartScore2; however, these changes were clinically relevant given their favourable direction, consistency, and relationship to cardiometabolic risk reduction.

**Clinical and standardized effect sizes at baseline and after 12-month training in total population and gender subgroups of overweight and obese adults in Poland (2023-2024)**

| Measure | N pairs for analysis | Baseline mean (SD) | 12-month mean (SD) | Absolute change | Relative change | Paired effect size $\boldsymbol{d}_{\boldsymbol{z}}$/ SRM | 95% CI for change | Interpretation |
| --- | --- | --- | --- | --- | --- | --- | --- | --- |
| Cooper test | 154 | 1502.8 (418.0) | 1983.8 (452.7) | +481.0 | +32.0% | **+1.45** | +428.2 to +533.8 | Large |
| BMI | 160 | 29.85 (2.77) | 29.20 (2.94) | −0.65 | −2.2% | **−0.38** | −0.92 to −0.39 | Small to moderate |
| Body Roundness Index | 148 | 4.85 (1.00) | 4.55 (1.10) | −0.30 | −6.1% | **−0.38** | −0.42 to −0.17 | Small to moderate |
| Glucose | 162 | 5.29 (0.54) | 5.04 (0.58) | −0.25 | −4.7% | **−0.53** | −0.32 to −0.18 | Moderate |
| Visceral fat rating | 145 | 10.48 (3.04) | 10.08 (3.10) | −0.40 | −3.8% | **−0.24** | −0.67 to −0.13 | Small |
| Insulin | 155 | 71.70 (36.56) | 61.17 (34.68) | −10.53 | −14.7% | **−0.35** | −15.35 to −5.71 | Small |
| HOMA-IR | 154 | 2.85 (1.59) | 2.34 (1.53) | −0.51 | −17.9% | **−0.38** | −0.73 to −0.30 | Small to moderate |
| Systolic BP | 161 | 132.9 (15.3) | 129.7 (14.9) | −3.26 | −2.5% | **−0.24** | −5.37 to −1.15 | Small |
| Diastolic BP | 157 | 87.7 (10.3) | 85.3  (9.9) | −2.36 | −2.7% | **−0.24** | −3.88 to −0.83 | Small |
| HeartScore2 % | 150 | 4.08 ± 2.55 | 3.59 ± 2.19 | −0.49 pp | −12.0% | **−0.41** | −0.68 to −0.30 | Small to moderate |
